# A Review of Recent Advances in Vital Signals Monitoring of Sports and Health via Flexible Wearable Sensors

**DOI:** 10.3390/s22207784

**Published:** 2022-10-13

**Authors:** Wenbin Sun, Zilong Guo, Zhiqiang Yang, Yizhou Wu, Weixia Lan, Yingjie Liao, Xian Wu, Yuanyuan Liu

**Affiliations:** School of Mechatronic Engineering and Automation, Shanghai University, Shanghai 200444, China

**Keywords:** wearable sensor, human movement, medical health, flexible electronics

## Abstract

In recent years, vital signals monitoring in sports and health have been considered the research focus in the field of wearable sensing technologies. Typical signals include bioelectrical signals, biophysical signals, and biochemical signals, which have applications in the fields of athletic training, medical diagnosis and prevention, and rehabilitation. In particular, since the COVID-19 pandemic, there has been a dramatic increase in real-time interest in personal health. This has created an urgent need for flexible, wearable, portable, and real-time monitoring sensors to remotely monitor these signals in response to health management. To this end, the paper reviews recent advances in flexible wearable sensors for monitoring vital signals in sports and health. More precisely, emerging wearable devices and systems for health and exercise-related vital signals (e.g., ECG, EEG, EMG, inertia, body movements, heart rate, blood, sweat, and interstitial fluid) are reviewed first. Then, the paper creatively presents multidimensional and multimodal wearable sensors and systems. The paper also summarizes the current challenges and limitations and future directions of wearable sensors for vital typical signal detection. Through the review, the paper finds that these signals can be effectively monitored and used for health management (e.g., disease prediction) thanks to advanced manufacturing, flexible electronics, IoT, and artificial intelligence algorithms; however, wearable sensors and systems with multidimensional and multimodal are more compliant.

## 1. Introduction

As the pace of social development accelerates, the demand for physical health monitoring has increased. In particular, since the COVID-19 pandemic, the focus on monitoring individual health has greatly increased, resulting in a significant increase in the demand for wearable devices. Although traditional health diagnostic and monitoring devices are effective, they have many limitations [1]. For example, devices used for real-time electrocardiogram (ECG) ambulatory monitoring have large sizes, many wires, and trigger skin irritation [2]. In addition, most traditional devices lack the portability to allow continuous, non-invasive health monitoring without interfering with daily activities. For older adults, it is inconvenient for them to travel back and forth to the hospital. This is also true for daily exercise. Especially, athletes need flexible wearable sensors that can be used to effectively monitor their training process without interfering with their training [3,4]. Advanced wearable sensors with flexible, remote, portable, and timely capabilities meet this demand and have applications in sports training, medical diagnostics, rehabilitation, and other fields.

Using flexible wearable sensors to monitor bioelectrical signals, motion information, and biochemical indicators is beneficial to personal health management. For this purpose, various flexible wearable sensors have been developed to monitor the above vital signals. For example, flexible wearable strain sensors are attached to the human body to monitor joint movement information, and inertial sensors are used together to improve training performances [5] and warn injury [6] during movement. Flexible wearable sensors can monitor the cardiovascular vital signs continuously and in real-time, including ECG [7,8], heart rate [9,10], blood pressure [11], blood oxygen [12], and blood glucose [13,14]. Advances in new materials, advanced manufacturing, and flexible electronics technologies have improved comfort, real-time, and precision and expanded the range of applications. Notably, with the help of the internet of things [15,16], big data technology [17,18], and artificial intelligence algorithm [19,20], the vital signals monitored via flexible wearable sensors can be used not only by the user to decrease damage during exercise, rehabilitation, and daily life, but also medical staff. In addition, the use of flexible wearable sensors reduces markedly the frequency of visiting hospital visits, saves hospitalization expenses, and effectively improves the medical experience and the allocation of medical resources.

**Figure 1:**
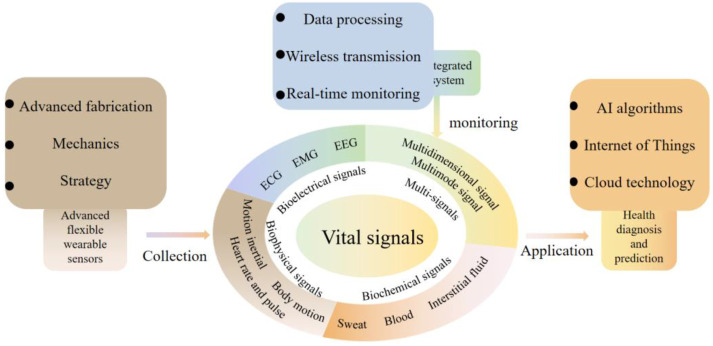
Processes for monitoring vital signals in health and exercise and an illustrated overview of this review. The article reviews each signal along the way process, including typical advanced wearable sensors, data acquisition and processing, and clinical and life applications.

Many current review articles have appeared on flexible wearable sensor-based sensors for health and exercise monitoring. However, when used for health monitoring diagnosis and prevention, it is difficult to rely on bioelectrical or physical signals alone for accurate diagnosis and assessment of diseases, and the same is true for sports training. For this reason, combining the analysis and summary of the current research, this paper classifies vital signals in sports and health as bioelectrical, biophysical, and biochemical signals, focusing on monitoring techniques using flexible, stretchable, and wearable sensors for monitoring these signals. More precisely, first, this paper reviews the monitoring of vital signals based on these three subfields, including ECG, electroencephalogram (EEG), electromyography (EMG), inertia, body movement, heart rate, blood, sweat, and interstitial fluid (Figure 1). For each signal, representative sensors for health and motion monitoring will be discussed in terms of sensing mechanisms, application needs, problem solutions, and technical features. Considering that single signals are difficult to be used accurately for health monitoring, this paper creatively proposes and reviews multidimensional and multimodal sensing devices and systems. This paper also summarizes the challenges, limitations, and future directions of wearable sensors for vital typical signal detection.

Through the review, the main contributions of the paper are: (1) the classification of vital signals in sports and health into bioelectrical, biophysical, and biochemical signals; (2) a review of representative sensors used for each signal monitoring in terms of sensing mechanisms, application needs, problem solutions, and technical features; (3) the first multidimensional and multimodal wearable sensing devices are presented and reviewed in detail; (4) the challenges, limitations, and future directions of wearable sensors are summarized.

## 2. Bioelectrical Signals

### 2.1. ECG

ECG is a momentous cardiac health indicator that contains a wealth of information about our cardiac state. Measuring ECG signals is one of the most common clinical exams. As the most typical wet electrode, Ag-AgCl is widely used in clinical practice. The electrolyte gel is used to decrease the contact impedance between the skin and the electrode. However, over time, the gel will become dehydrated, leading to increased contact impedance with the skin and skin irritation [21]. While dry electrodes have been developed to overcome these defects and get high-quality signals, motions and skin deformations can generate air gaps between the skin and the electrode, resulting in increased impedance and motion artifacts. A novel, wearable, and flexible dry ECG detection platform based on different detection methods have been developed for real-time ECG monitoring. Specifically, ECG signals are detected in both contact and non-contact ways.

**Figure 2:**
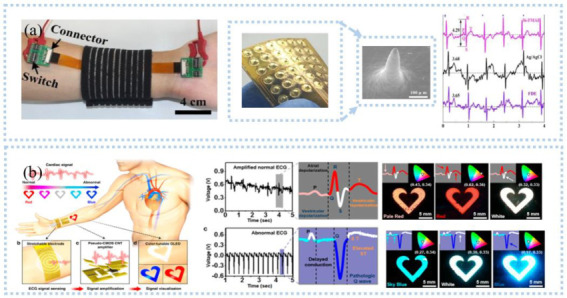
ECG signal detection: (**a**) ECG sensor with microneedle electrodes collected ECG at the wrist and compared with wet electrodes and dry electrodes. Reprinted with permission from Ref. [26]. Copyright 2017 Elsevier, (**b**) organic light−emitting device used for ECG monitoring and the collected ECG was visualized. Reprinted with permission from Ref. [36]. Copyright 2017 American Chemical Society.

Wearable sensors containing detection circuits for accurate and real-time ECG monitoring require reliable connectivity, comfort, and high signal transmission quality. An example is all-in-one, wireless, stretchable hybrid electronics [22]. The thin-film electronic layers and hyperelastic elastomers allowed the device to cling tightly to the skin and stretch as the skin deforms. The electrodes consisting of circular islands and meander lines allowed for stretchability and conformal contact to the skin, obtaining clinical-grade ECG. Excellent permeability is important for long-term monitoring. This structure allowed the electrode to have excellent permeability and evade the effects of sweat, particularly during movement [23]. Excellent permeability can also be obtained by introducing pores on the substrate-free electrode [24]. For simultaneously satisfying high conductivity, compliance, conformal coverage contact area, and detection of weak ECG signals, Asadi et al. [25] proposed sponge-structured graphene electrodes with high compressibility (85%) and conductivity. High compressibility reduced the gap between the skin and the electrode and improved the conductivity. Without filtering, QRS points can be easily obtained, even under motion artifacts. Unlike patch electrodes, microneedle electrodes can obtain high-quality electrical signals at the nerve endings through the stratum corneum. Ren et al. [26] prepared a flexible microneedle array electrode on a flexible substrate (Figure 2a) by novel magneto-rheological drawing lithography for ECG detection in static (lying flat) and dynamic (running) processes. The microneedles penetrated the corneum [27] without pain under 1N force, reducing the impedance of the corneum to 104 kΩ (50 Hz) and collecting the ECG signal with low attenuation.

In contrast to contact ECG detection, capacitive sensors can extract the ECG signal by capacitive coupling without contact with the skin. However, they are unsuitable for real-time ECG monitoring, especially in sports. That is because non-contact ECG detection is susceptible to motion artifacts. To this end, Gao et al. [28] proposed a non-contact ECG monitor for real-time ECG monitoring, consisting of flexible capacitive electrodes and detection and amplification circuits. The copper protective rings were placed around the electrodes and fixed to the skin with conductive tape to reduce the effect of motion artifacts. A novel motion artifact removal adaptive filtering method for capacitive ECG sensors was proposed [29]. The reference signal reflecting the capacitive change can be extracted by modifying power-line interference for adaptive removal of motion artifacts. Then, a special blind source separation algorithm was used to remove the moving artifacts and extract fetal ECG [29]. Taking a different approach, wristbands or chest bands that integrate capacitive sensors and signal processing circuits have stable contact with the skin by the force exerted by the elastic band to reduce the influence of motion artifact [30], which means that smart clothing can be developed for real-time ECG monitoring, as demonstrated by the study of Nemati et al. [31].

ECG contains a wealth of information related to heart health and is widely used for heart disease monitoring and prediction, such as acute myocardial infarction [32], sepsis onset [33], and stroke [34]. To obtain ECG in real-time and remotely, an IoT-based wireless communication system is needed to transmit the collected heart to a smart terminal. A Bluetooth-based electronic system, including an onboard Bluetooth module, a data processing module, and a sensing module, was developed to monitor ECG and respiratory rate in real-time in a wireless manner [22]. The data is transmitted to a smartphone via Bluetooth and processed by classification and feature extraction to display the child’s ECG, heart rate, and respiration in real time. This device was useful for pediatric care. With the help of AI algorithms, personal health can be effectively monitored by analyzing the data in real-time. For example, Hussain et al. [35] proposed a cyber-physical cardiac monitoring system for stroke management. The system contained a wearable ECG sensor, data storage, and data analysis. They extracted electrocardiograms monitored by wearable devices to obtain biomarkers associated with stroke and used supervised machine learning techniques to classify strokes. The system was found to be effective in identifying stroke categories with an overall accuracy of 95.6% under a random number model, which is important for prognosis and rehabilitation management during post-stroke treatment. Although there is a great development of wearable sensors and systems for ECG monitoring, however, patients or users are not able to analyze the obtained ECG signals effectively. In addition, senselessness, lightweight, and high flexibility are important for wearables, especially during exercise. Visualization is a major direction for ECG monitoring systems in the future. Current visualization is still based on rigid hardware, such as displays. In contrast, Koo et al. [36] developed a flexible wearable heart monitor composed of a snaky-shaped gold (Au) thin-film ECG sensing electrode, a *p*-MOS CNT signal amplifier, and a color-tunable organic light-emitting diode (CTOLED), as shown in Figure 2b. CTOLED displayed light red, red, and white when detecting a normal ECG signal and white, sky blue, and dark blue when detecting an abnormal signal. In addition, the ECG monitoring sensor or system should have closed-loop monitoring, such as an early waring function.

### 2.2. EEG

**Figure 3:**
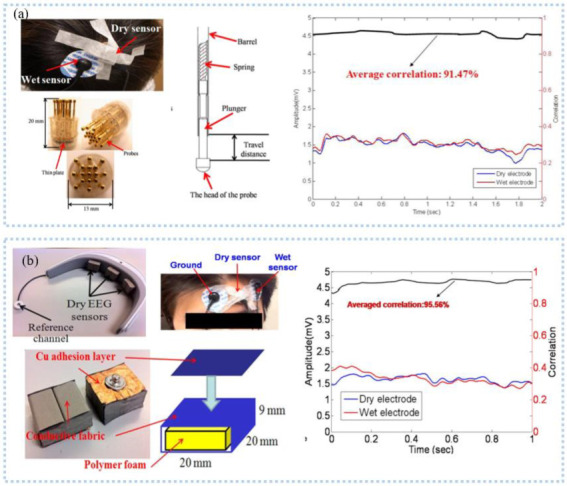
EEG signal detection: (**a**) spring electrode for monitoring EEG in the presence of hair, and had a high agreement with the wet electrode. Reprinted with permission from Ref. [38]. Copyright 2011 MDPI, (**b**) a wearable EEG acquisition device with a dry foam EEG sensor for collecting EEG at the forehead. Reprinted with permission from Ref. [45]. Copyright 2012 Springer Nature.

Several studies found that hair interference must be settled when EEG is detected in areas with hair. To solve this problem, needle-like electrodes have received a lot of attention in EEG detection. The hairs reduce the contact area between skin and electrode, but needle-like electrodes can remove the hairs to directly contact with skin [37]. From this, Liao et al. [38] proposed a spring-contact probe structure electrode (Figure 3a) with an impedance comparable to the wet electrodes and even lower at the root of the hair to detect EEG, which can achieve a high signal quality even during motion [39,40]. Then, Ren et al. [41] fabricated PDMS-based columnar microneedles, and connected the electrode with a headset, which exhibited a high-quality EEG signal comparable to the wet electrode and less variation in prolonged EEG detection. The PDMS columnar array helped microneedles to pass through the hairs and the tips of the microneedles to pass through the corneum into the epidermis, reducing the electrode-skin interface impedance. Another popular device for detecting EEG is a capacitive sensor. A non-contact capacitive sensor with common mode noise rejection was developed by Chi et al. [42], with a noise level RMS 3.8 μV and a gain of 46 dB ranging from 0.7 to 100 Hz and applied in detecting EEG signal. After, the EEG data was digitized, amplified, and filtered by data processing circuits and transmitted to the intelligent device through wireless communication for visualization and continuous monitoring, which can significantly improve the comfort of the user. Then, they integrated the sensor into the fabric and developed a wireless, non-contact headband for EEG monitoring [30]. The electrodes were placed on the forehead and back of the brain and can acquire clear α waves of brain activity comparable to the wet electrodes.

As we know, the wearable EEG sensor that takes the form of a headband can maintain stable contact with the skin due to a fixed force applied by the elastic band. Carneiro et al. [43] printed a silver base conductive fabric electrode and developed an EEG headband, which was connected with a rigid PCB island and placed on the forehead to detect EEG. The short distance between the electrode and the amplifier circuit can significantly reduce the noise caused by the long wire, and the second layer of contact electrode used to connect the rigid PCB island can reduce electromagnetic interference. After amplification, digitization, and filtering, the EEG data was transmitted to the smart terminal using WiFi. To meet the demand to protect user privacy, Goverdovsky et al. [44] integrated conductive fabric and a viscoelastic substrate to develop the earplug structure electrode, which was made by sewing stainless steel threads onto silver-plated fabric and used to detect EEG inside the ear canal in an imperceptible way. The viscoelastic substrate allowed the electrode to have stable contact with the skin, and the conductive fabric has a low impedance with the skin after being wet with a salt solution. Comfort is necessary for long-term EEG monitoring. Liao et al. [45] proposed an EEG sensor, consisting of polymer conductive foam, conductive fabric, and copper adhesive layer (Figure 3b). The device can adapt to irregular skin, and the conductive foam ensures comfort during long-term EEG monitoring. Li et al. [46] printed the Ag/AgCl dry electrode that included a sponge layer with water absorption. The sponge layer reduces the electrode impedance with the skin by absorbing sweat while avoiding crosstalk and short circuits between adjacent electrodes caused by sweat.

EEG records the electric action of neuronal groups and provides vital information about cerebral diseases such as stroke, mental workload, and stress [47,48]. In one study, researchers developed a real-time health monitoring system for stroke prognosis that incorporates an embedded eye-patch portable EEG device and data analysis [49]. Stroke markers brain symmetry index, δ-α ratio, and δ-θ ratio were extracted and support vector machine models in machine learning analysis were used to classify strokes, which can be as accurate as 92%. Then, they used this study in an advanced driver assistance system. The binary machine learning classification model showed near-perfect accuracy between resting and driving states. In addition, the event-related potentials can be acquired by examining brain activity and used to analyze the perception and cognitive activity [48]. Hussain et al. [50] used wearable devices to monitor cardiovascular and neurological response markers during microwave brain stimulation. It was found that cognitive workload and heart rate variability could be effectively differentiated by recording the initial resting state, intermediate state, and final state EEG and ECG. Subsequently, they used EEG to study the neurological changes induced by sleep stages [51]. Quantitative neuro-EEG biomarkers of fast waves α, β, and γ and slow waves δ and θ could be used to differentiate sleep stages by supervised data analysis of the C5.0 model with 91% accuracy.

From the above, it is clear that EEG can be effectively monitored by wearable sensors, and machine learning algorithms and deep learning algorithms can be used for disease prediction and stress analysis, among others. It is important to note here that EEG monitoring systems must be commercially available. However, miniaturization and personal privacy severely limit the use of EEG monitoring devices for commercialization because people do not want to be perceived as having a disease. Secondly, the relationship between EEG monitoring devices and body-worn aesthetics should also be looked at so that they will be accepted by the general public.

### 2.3. EMG

**Figure 4:**
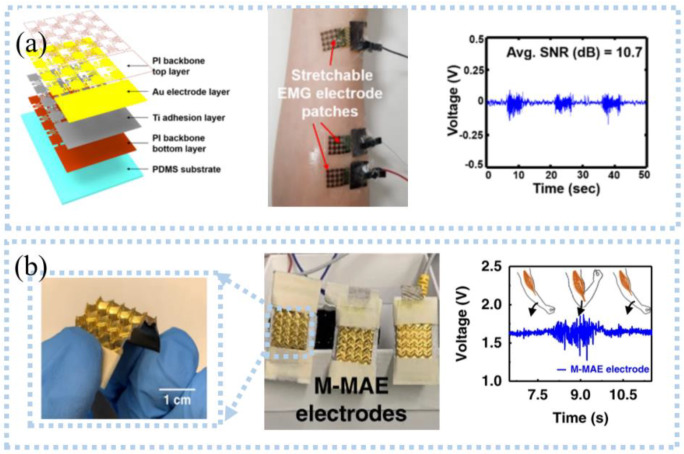
Detection of EMG signals: (**a**) photographic image of the ultrathin, stretchable electrode patch (left), wireless epidermal EMG sensing system (middle), and EMG signals in clenching a fist motions (right). Reprinted with permission from Ref. [52]. Copyright 2020 MDPI, (**b**) pyramid—microneedle electrodes (left), EMG acquisition device with M−MAE (middle), and EMG signals for two arm–bending cycles (right). Reprinted with permission from Ref. [58]. Copyright 2020 Springer Nature.

Similar to ECG and EEG, EMG is allowed to be detected with or without skin connection. Currently, EMG sensors need to be self-adhesive, flexible, and stretchable to overcome slippage and maintain stable contact with the skin to obtain high-quality EMG signals. For example, Lee et al. [52] developed an ultra-thin Ti/Au snake-like electrode on PDMS substrate (Figure 4a), which can stretch as the skin deforms during movement, and the maximum SNR was 15.77 dB. The electrode adhered strongly to the skin, and no slippage occurred when EMG signals were measured during leg lifting. After being processed by the data processor, the data is transmitted wirelessly via Bluetooth to the computer side. The system can effectively assess the muscle weakness of elderly and disabled people in their daily life. Unlike skin contact, capacitive electrodes use a dielectric layer to monitor the EEG without direct contact with the skin. Liu et al. [53] proposed a non-contact EMG acquisition system. Flexible non-contact electrodes comprised of a two-layer FPC were connected to the main PCB board using a soft shielded cable to detect EMG. They placed a copper shielding ring on the front of the electrode and a copper shielding layer on the back to remove various environmental interference. The collected EMG was transmitted to the PC via a Wi-Fi network after buffering, amplification, and digitization. The system can effectively distinguish between an open or closed hand as the electrodes were placed on the arm. Since then, they added a band-pass filter to filter motion noise and found that the electrode showed an excellent performance, which was evidenced by the high-quality EMG signals obtained during the opening or closing of the upper arm muscles [54].

However, several obstacles must be addressed in the EMG monitoring process, such as motion artifacts, sweat, and high impedance [55,56]. For example, epidermal surface lipids contaminate the electrode, resulting in poor contact between the electrode and the skin, a potential barrier, and a lower SNR. He et al. [57] proposed an on-skin electrode with anti-epidermal-surface-lipid function via grafting a zwitterionic polymer on top of gold-coated (Au/PDMS), which can be cleaned by water flushing, maintaining a stable impedance with skin and high SNR. EMG can also be detected by the microneedle electrode, which can improve the selectivity and eliminate crosstalk [58]; the pyramidal microneedle electrode (Figure 4b) was developed for detecting EMG [59]. The microneedles reduced motion artifacts by breaking through the corneum barrier and forming stable contact with the skin. The pyramid structure created air gaps between electrodes and skin, allowing air to flow through pyramidal microchannels to remove sweat. For motion artifacts, filtering or Fast Fourier Transform can quickly pre-process the acquired data to remove motion noise. Unlike these, Yun et al. [60] reduced motion artifacts by depositing gold nanoparticles on flexible substrates. These nanoparticles improved the surface area of the electrodes, which significantly reduced interfacial impedance and thermal noise, and motion artifacts were reduced by 95%. In another case, flexible electrodes and signal processing circuits were integrated onto a wristband to classify the EMG signals [61]. The device had stable contact with the skin and minimized motion noise by applying pressure. Posada-Quintero et al. [62] prepared a strip EMG electrode from the mixture (CSA) of toner, salt, and viscoelastic polymer adhesives, which needed to be activated by applying a certain voltage before use. It was concluded that the carbon pillars formed in the Z direction during activation can resist skin movement while being less sensitive to the potentials in the X and Y directions, thereby eliminating the effects of motion artifacts. Compared with commercial wet Ag/AgCl electrodes, these electrodes had better SNR (38.3 dB) and signal-to-motion ratio (24.1 dB). The same group also investigated the use of CSA electrodes for detecting EMG and found that CSA electrodes can be better to identify subtle changes in EMG signals caused by muscle fatigue than wet electrodes [63], which can be applied to monitor the progression of muscular dystrophies or control muscle activities in the future.

EMG contains the subtle changes in the electrical signal caused by muscle excitation, which has been applied in sports and neuromuscular rehabilitation. For example, to assess stroke-damaged muscle activity, an EMG-based gait monitoring system was developed to monitor EMG biomarkers [64]. The system performs feature extraction of mean work frequency, median work frequency, peak work frequency, and mean power in EMG of biceps femoris and bilateral lower extremity lateral gastrocnemius muscles, followed by classification of stroke patients and healthy adults using neural network models in machine learning algorithms with an accuracy of 80%. However, EMG or motion sensors alone are difficult to use for muscle status monitoring [65]. Park et al. [66] combined EMG signals with plantar pressure signals to obtain gait, an important marker of disability, injury, and gait symmetry. This is followed by the prediction of gait disorders and healthy gait using IoT and machine learning algorithms. Another case is the study by Di Giminiani et al. [67]. They proposed a smart clothing system by combining fabric EMG sensors and oximeters for monitoring quadriceps muscle activity during training. Compared with a commercial gold-standard EMG and oximetry system, they found that the system had high reliability and the limited stretching of the fabric electrode led to motion artifacts and reduced signal quality, particularly during motion.

EMG monitoring has high requirements for muscle position, i.e., it requires the user to wear it exactly in the specified position. However, traditional EMG monitoring devices often require specialized personnel to wear them properly. In the future, it is hoped that EMG monitoring devices can be developed that are easy to install and wear. In addition, miniaturization and senselessness are also required, which is important in sports and post-operative rehabilitation.

## 3. Biophysical Signals

### 3.1. Motion Inertial

Motion inertial contains a large amount of kinesiology and kinematic mechanics information, which have been applied in assessing motion quality and injury prediction. For example, inertial motion information can be used to assess the risk of falling. Effectively monitoring, analyzing, and deciding on this information is vital for medical diagnosis [68], rehabilitation, and sports and special training [3]. Stair climbing is part of training, military training, and fitness. In one case, Ojeda et al. [69] taped inertial sensors to the feet to obtain the movement data during the training of climbing stairs and found that the risk of tripping/falling and damage from ground forces and bounce angles can be assessed by analyzing the gap between the feet. Marks, non-portable, and high costs limit the use of optical motion tracking systems and video motion tracking systems, which are the gold standard of motion monitoring. However, inertial sensors eliminate these limits by wearing them directly on the body. Some studies found that the inertial sensors had a high consistency (>0.9) with the gold standard [70]. Hence, the motion information obtained by the inertial sensors during training can be applied to evaluate the performances of athletes in training, and provide actionable data for athletes, coaches, and researchers to improve training plans, and equipment.

**Figure 5:**
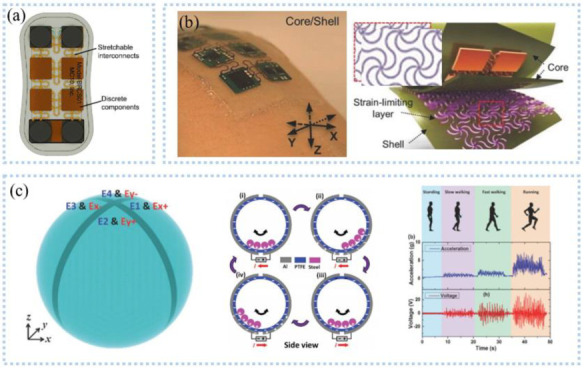
Inertial signal detection: (**a**) bottom view of motion sensing patch. Reprinted with permission from Ref. [71]. Copyright 2020 John Wiley and Sons, (**b**) optical images and an exploded view schematic illustration for a core/shell structure. Reprinted with permission from Ref. [72]. Copyright 2015 John Wiley and Sons, (**c**) schematic diagram showing the T−ball device structure (left), operation mechanism of the T−ball under in−plane vibration along x−axis (middle), and the acceleration level and the output voltage from different type of exercises (right) Reprinted with permission from Ref. [73]. Copyright 2017 John Wiley and Sons.

To get rid of the discomfort caused by rigid inertial sensors, Ammann et al. [71] implanted accelerometers and gyroscopes into the skin adhesive patch to develop a wireless and wearable motion tracking patch, as shown in Figure 5a. They used the device to track the movement of the arms, and the obtained data was transmitted to a computer through Bluetooth. After data analysis, the limb movements could be accurately assessed with a consistency of 0.95 with the video motion tracking system. Integrating accelerating sensors that were connected to snake-like stretchable wires on a flexible substrate (Figure 5b), Lee et al. [72] proposed a stretchable inertial tracking system to capture various body motions. The PI network embedded between the Ecoflex as the outer layer and silicone as the inner layer limited the strain to as much as 20% to protect the device. After encapsulating with a wireless communication device, the device can be securely attached to the human skin and successfully identify the movements of lifting dumbbells without falling off. These studies have improved comfort and adaptability through the use of flexible packaging. Unlike using inertial sensors to track motions, Shi et al. [73] prepared a gyroscope ball (Figure 5c) based on a 3D symmetrical triboelectric nanogenerator (TENG) to monitor the inertial information. The device simultaneously detected multi-axis acceleration and ration and was capable of collecting energy. They found that the output voltage of Ex corresponds with the magnitude of acceleration during moving when the x-direction of the device was consistent with the moving direction, which can identify standing, walking, walking fast, or running slow. However, inertial sensors based on self-power need an external monitoring circuit. To decrease complexity and improve stability and sensitivity, Xie et al. [74] proposed a gyroscope based on the impedance matching effect of TENG for measuring relative rotation angle, containing a freestanding-mode rotary dis-shaped triboelectric nanogenerator, a resistive rotation angle sensor, and a light-emitting diode (LED) alert display, with a high sensitivity of (67.3 mv^−1^), good linearity between the output voltage and the rotation angle, and quick response of 20 ms in the range of 0~260°. At the same time, the device removed the batteries and management circuits to simplify the system and used the quantized LED to display the rotation angles.

With the increase in age, older people are more likely to fall accidentally, which can lead to accidental death. To this end, inertial sensors were placed on the human body to collect movement data, and the motion noise was removed by a filter. After the data is transmitted via wireless communication to a computer and analyzed by a special algorithm, it can be found that the inertial sensors can detect falls. Good sleep quality is important to human health, and the magnetometer sensor has been used to detect the subtle changes in magnetic vectors to millimeter-scale respiratory movements during the night [75], which can be used to assess sleep quality after processing by intelligent algorithms. Multiple sclerosis is a common neurodegenerative disease in which balance disorder is one of the symptoms and can be diagnosed by posture detection. Sun et al. [76] placed a BioStamp wireless inertial sensor on the L5 back of the torso, near the COM of the body, to record posture swings while standing. Compared with the gold standard, it can be concluded that wearable inertial sensors are a promising choice for diagnosing multiple sclerosis.

### 3.2. Body Motion

**Figure 6:**
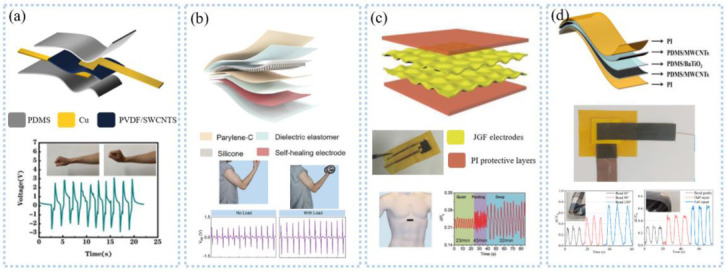
Body motion detection: (**a**) piezoelectric sensors for monitoring wrist movements. Reprinted with permission from Refs. [77]. Copyright 2021 Elsevier, (**b**) tribological sensor for monitoring upper arm muscle action. Reprinted with permission from Refs. [78]. Copyright 2021 American Chemical Society, (**c**) piezoresistive sensor for monitoring breathing. Reprinted with permission from Refs. [79]. Copyright 2018 John Wiley and Sons, and (**d**) capacitive sensor for monitoring joint action. Reprinted with permission from Refs. [80]. Copyright 2021 MDPI.

While the wearable-based inertial sensor exhibits high precision for body motion detection, motions such as bending joints and swallowing cannot be detected by using it. It contains a large amount of health information and has been applied to improve training performance and diagnose Parkinson’s disease, Alzheimer’s disease, and diabetes. Wearable sensors based on various mechanisms such as piezoelectricity [77], triboelectricity [78], piezoresistance [79], and capacitance [80] have been proposed to monitor body motions, as shown in Figure 6. These sensors exhibit excellent performance, containing high sensitivity, durability, and flexibility for monitoring body motions, such as finger bending.

Wearable strain sensors attached to the skin with patch structure deform along with a body motion to detect joint bending [81,82]. For example, Zhao et al. [83] developed a PVDF-based sensor, which uses piezoelectric output as a sensing signal. To analyze the performance of playing basketball, they attached the sensor to the joint and applied the multi-point control function to monitor the sequence of the change of force order, angle, and motion frequency. Nevertheless, strain sensors are easily affected by motion artifacts, resulting in a reduction in measurement precision. This problem can be solved by endowing the substrate with self-adhesive and elastic properties. Wang et al. [84] developed stretchable, drying, and self-adhesive strain sensors to detect the motions of the ankle, wrist, and neck. The dry adhesive layer made of waterborne polyurethane allowed the sensor to stick tightly to the skin and has a high sensitivity (GF = 89), while the motion artifacts were significantly diminished. In the long run, the sweat generated will cause discomfort to users and reduce the precision of the measurement. Bi et al. [85] used conductive fabric to monitor the motions of limbs. The conductive fabric was prepared by coating modal/spandex fabric with rGO/carbonated ink/PVA and attached to the wrist, elbow, knee, and ankle to effectively monitor the joint bending, which can be used to correct the posture of professional athletes, such as basketball or badminton players, after analyzed by coaches or specialized algorithms.

Vital health information can be obtained by monitoring body motions. A highly stretchable transparent wearable soft ion skin system was developed [86], containing a 10-channel hydrogel/elastomer hybrid ion sensor and a wireless electronic control module. The ion sensor was prepared by solidifying hydrogel onto a preformed elastomer treated with benzophenone to form a hydrogel-elastomer hybrid structure, with high scalability (300%) and transparency (95%). The system was fixed on the hand to identify various movements and sent to a smartphone through wireless communication, giving the sensor the ability to recognize sign language to help the deaf and speech-disabled communicate. Conductive fabrics retain comfort and softness fabric, which is important for users who need to wear them for a long time, especially patients, as demonstrated in the study done by Zhao et al. [87]. In addition to monitoring the motions of joints, wearable strain sensors also have been applied to detect the motions of feet, which are widely used in feet type assessment, sports training, clinical gait analysis, and feet pathology diagnosis. To collect gait information in real-time, Mao et al. [88] proposed a self-powered portable TENG. Using 3D printing technology, the performance of the sensor was significantly improved as demonstrated by the results. They embedded the sensor in sneakers to monitor gait and stability during various movements, such as in-situ movements and walking. Piezoresistive sensors based on liquid metal were also used to measure plantar pressure. The sensor used eutectic gallium indium (EGaIn) filled in the 3D-printed ABS spiral pattern microchannel as conductive liquid and can detect the pressure of plantar and ankle from 0 kPa to 400 kPa [89]. Data acquired by the sensors and flexible circuits were integrated into the insoles and transmitted via Bluetooth and analyzed by a special algorithm. The result suggested that the sensor can successfully distinguish between walking and running movements by analyzing weight during the posture and swing phases. Besides the pressure insole, the smart socks consisting of TENG, signal preprocessing circuit, and microcontroller with wireless transmitter used the triboelectric output voltage as a pressure signal to monitor the plantar pressure, which was analyzed by the convolutional neural network algorithms to obtain the gait and monitor personal health [90].

Further work is to optimize the flexibility and portability of wearable sensors. With the development of flexible electronics, wearable sensors containing detection circuits can be integrated on flexible substrates and worn on the body to realize real-time detection of movements. For the above wearable sensors, motion artifacts still bother them. At present, motion artifacts can be removed by designing special algorithms and adding filters. A self-adhesive substrate is also a good choice, allowing the wearable sensor to stick tightly to the skin. In particular, the substrate made of skin-like materials can co-form with the skin without creating air gaps. The development of hydrogel has solved the problem that the conductive material cannot be deformed on a large scale and can be directly attached to the skin, with both viscosity and excellent tensile properties. In addition to the interference of motion artifacts, electromagnetic is also one of the interference sources in motion detection. The sensor based on optical fiber can overcome electromagnetic interference well and is less often investigated than the sensors mentioned above but can also be found in the literature. For example, Leber et al. [91] developed a stretchable strain sensor for bending motion detection, such as knee joint and finger motion, by embedding optical fiber into cloth.

### 3.3. Heart Rate and Pulse

Heart rate and pulse are two simple indicators of cardiovascular health, which are often measured by sensors placed on the chest, wrist, neck, and fingers. To accurately monitor cardiovascular health, electrical sensing, pressure sensing and, optical sensing has been developed to extract heart rate and pulse. Using electrical sensing to detect the electrical activity of the heart was found in several studies. For example, Rodeheaver et al. [92] extracted the heart rate by peak-seeking and window averaging of ECG. Kim et al. [22] developed a scalable hybrid electronic system consisting of three electrodes, flexible circuits, motion sensors, and wireless emission modules that enable real-time and remote monitoring of ECG, heart rate, and respiration.

**Figure 7:**
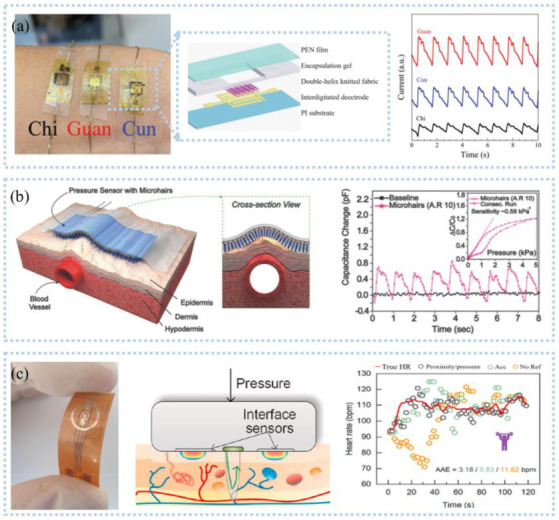
Heart rate and pulse detection: (**a**) The 1 × 3 sensor array for pulse detection (left), the twisted yarn with a double−helix configuration (middle), and pulse on the position of “Cun”, “Guan”, and “Chi” (right). Reprinted with permission from Ref. [94]. Copyright 2021 John Wiley and Sons, (**b**) Schematic illustration to detect pulse (left) and the sensitivities (right). Reprinted with permission from Ref. [97]. Copyright 2015 John Wiley and Sons, (**c**) A photograph of the interface sensor (left), the principle of interface sensor for perceiving contact pressure on the skin (middle), and HR estimation results for weightlifting (right). Reprinted with permission from Ref. [98]. Copyright 2021 Elsevier.

Unlike the heart rate from the ECG, the pressure sensor can get the heart rate and pulse directly. Rasheed et al. [93] prepared a pressure sensor by utilizing a piezoelectric charge-gated thin-film transistor made of PVDF piezoelectric sandwich structure and amorphous silicon double-gate TFT as the main components, which can simultaneously detect the heart rate at multiple points. Ultra-thin PZT and semiconductor materials were integrated on silicone thin elastic material for skin pressure monitoring with an extremely low detection limit (0.005 Pa) and rapid response (0.1 ms) for arterial pulse detection at the wrist and neck. Besides the piezoelectric sensor, Chen et al. [94] used twist technology to prepare an ultra-fast response/recovery flexible piezoresistive sensor for pulse detection, as shown in Figure 7a. Twisting technology enables the sensor to have a DNA double helix structure, which makes the fibers tightly stacked, giving the sensor a fast response and low lag. To measure the pulse at high sensitivity, Nie et al. [95] proposed a capacitive sensor based on ionic droplets, which can detect the carotid pulse at a high sensitivity of 1.85 uF/kPa. After that, they improved the sensor sensitivity to 0.43 nF/kPa by developing an array of ion droplets and successfully detected the arterial pulse at the wrist [96]. In addition to measuring arteries, the pulse of deeper veins is also used to assess cardiovascular health but is difficult to measure. Pang et al. [97] proposed a capacitive sensor with a micro-brush structure (Figure 7b). The micro-brush structure improved the effective contact between the sensor and irregular skin, achieved super-consistency between the sensor and the skin, and improved the SNR by 1-2 imes. The signal amplification function of the micro-hairs enabled the sensor to detect the weak pulsation of the internal jugular vein pulse (Figure 7b).

Photoplethysmography (PPG) detects volume changes in blood flow through the skin to obtain heart rate information. Current commercial equipment based on PPG is large in size and high in power consumption, and it is hard to extract accurate heart rate and pulse information in the presence of strenuous exercise or optical noise. Motion artifacts are mainly derived from the interface dynamic changes between wearable sensors and body skin; Scardulla et al. [9] studied the contact pressure between PPG sensors and the skin and found that the influence of contact pressure was greater than that of exercise. The Pearson correlation coefficient was 0.81~0.95 when the contact pressure was 54 mmHg, and the average percentage error range was 2.4%~3.8%. Wang et al. [98] proposed a PI-based interface sensor consisting of a platinum film thermistor and a reflective PPG sensor (Figure 7c) to detect the contact pressure between the sensor and the body skin. The thermistor distinguished pressure by detecting the interface temperature field between the sensor and the skin. The sensor can be fitted on any irregular surface, enabling accurate detection of heart rate during exercise such as weightlifting (Figure 7c). The organic photoelectric sensors are manufactured directly on a flexible substrate to make a flexible PPG sensor, such as the organic photodetector based on non-fullerene receptors proposed by Simões et al. [99], which can be adapted to the skin to detect heart rate. In the detection of deep arteries or blood vessels, near-infrared light is more advantageous than visible light because of its longer wavelength, resulting in greater penetration depth and fewer optical attenuation. Sensors with ultra-thin structures allow flexibility, comfort, softness, and mechanical compatibility to be retained, which is beneficial for long-term monitoring. Chen et al. [12] developed a multiwavelength array of flexible PPG sensor patches for detecting pulse information during sleep, walking, and cycling. The array design decreased the effect of motion artifacts, resulting in a heart rate accuracy of 92% and blood oxygen accuracy of 95%.

Heart rate variability contains information on the regulation of the cardiovascular system by neurohumoral factors and is often used in the diagnosis and prevention of cardiovascular disease and in the detection of mental stress. In one case, Correia et al. [100] extracted HRV from PPG and ECG signals at rest and by performing a stress-inducing task: the stroop colorimetric test. The consistency of HRV was determined by calculating RR and PP intervals and successfully diagnosing stressors. Another example is the study done by Pramukantoro et al. [101]. They achieved classification of heartbeat by extracting heart rate abnormality feature RR interval. The oversampled data can achieve 99.67% automatic correct classification of heartbeats after processing by the random deep forest model in machine learning. This is important for early monitoring of cardiovascular diseases. The majority of current heart rate monitoring devices are smart wristbands, which are comfortable, convenient, and inconspicuous to wear. While some research is turning to smart clothing, it is important to note that it must have the advantages of a smart wristband. Second, smart clothing must also have a high degree of monitoring accuracy, which is important for disease prediction.

## 4. Biochemical Signals

### 4.1. Sweat

**Figure 8:**
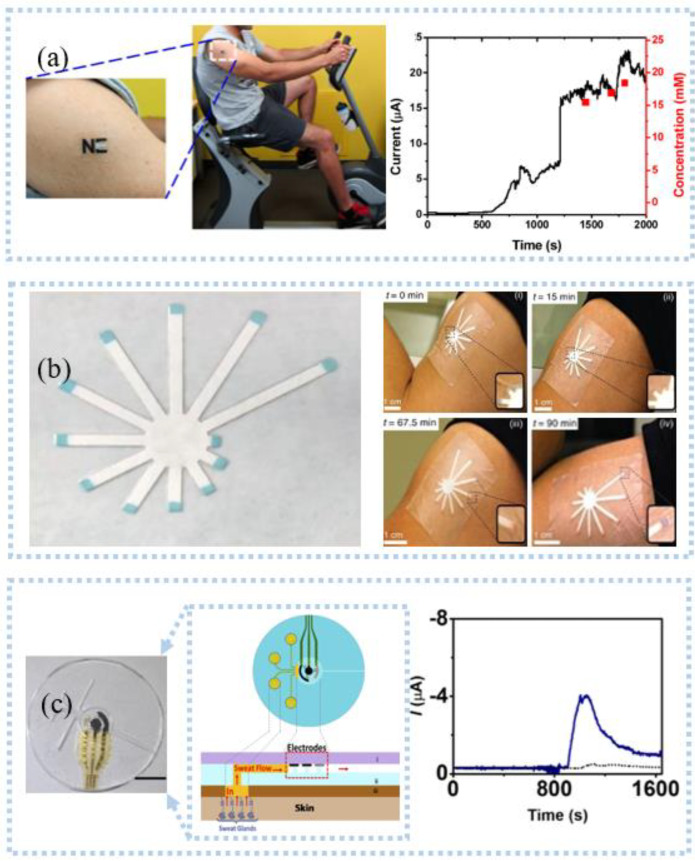
Sweat detection: (**a**) an electrochemical electronic tattoo applied to detect deltoid and real−time response and corresponding lactate concentrations during a cycling exercise. Reprinted with permission from Ref. [104]. Copyright 2013 American Chemical Society, (**b**) photo of paper colorimetric sensor and the sweat patch being used on the upper arm of a person working out. Reprinted with permission from Ref. [113]. Copyright 2019 Springer Nature, (**c**) Schematic representation of microfluidic device sweat collection and operation (left), physical integrity analysis of microfluidic platform (middle), and continuous lactate monitoring (right). Reprinted with permission from Ref. [117]. Copyright 2017 American Chemical Society.

As one of the most important fluids in the human body, sweat contains a wealth of information related to human health. Sweat detection sensors based on different detection principles have been developed to enable continuous in-situ sweat analysis. For example, Sempionatto et al. [102] prepared an electrochemical sensor for the detection of lactic acid, glucose, and potassium ions by screen printing, which was integrated into eyeglasses. The device transmitted the data to the host device through Bluetooth to realize the real-time detection of markers in sweat. The detection of ions in sweat is important for the human body and can distinguish between aerobic and anaerobic exercise. Commercial carbon fibers modified with Na^+^ selective membrane can be selectively detected with high accuracy (55.9 ± 0.8 mV/log [Na^+^], N = 3) for Na^+^ in the range of 10^−3^ M~10^−1^ M [103] and integrated into textiles to develop smart clothing. Jia et al. [104] prepared an electronic tattoo wearable sensor (Figure 8a) that consists of molecularly printed polymers and silver nanowires. The sensor can detect lactic acid with high sensitivity at 0.22 μM. Wearable flexibility is the demand of the future; Zamarayeva et al. [105] utilized flexible printing to create paper-based electrochemical sensors. The carbon nanotube (CNT) layer between the film and the reference electrode was used to adsorb Cl^−1^, resulting in a more stable reference electrode. The PVC film with the restriction limited the diffusion of lactic acid, making the detection of lactic acid not affected by sweat flow and improving the sensitivity (3.28 μA/mM). Moreover, paper-based sweat sensors also can be disposable components [106].

In addition to electrochemical sensors, wearable sensors based on optical sensing are used to detect sweat. An optical sensor based on (2-hydroxy-1,4-naphthoquinone or Lawsone) HNQ was used to detect sodium ions, pH, and urea in sweat [107]. Sodium ions affected the absorbance of HNQ, hydrogen ions reduced the reactivity of HNQ, and urea promoted more interaction of sodium and potassium ions with HNQ. As a common optical sensor, the colorimetric sensor for sweat detection has the characteristics of visualization. For example, Zhou et al. [108] developed a sweat sensor based on gold nanoparticles (AuNPs) colloids, which can quickly distinguish dehydration from overhydration by observing color changes. Fabric-based colorimetric sensors are used for pH and lactic acid detection [109]. Sweat pH was measured using a mixture of methyl orange and bromocresol green, and lactic was determined using lactic. In the future, sensors could be integrated into wearable fabrics to make clothing with smart sensing. The colorimetric sensor consisting of cotton thread and functionalized filter paper had a wide dynamic detection range (50~250 μΜ) and a low detection limit (35 μΜ). After integrating with an armed guard for sensitive detection of glucose in sweat, the sensors had the advantages of low cost and ease of use with the aid of a smartphone [110]. Ardalan et al. [111] developed a fluorescence-based wearable patch consisting of filter paper, cotton thread, and medical tape for the detection of glucose, lactate, pH, and chloride. A 3D-printed imaging module was equipped with ultraviolet (UV)-LED lights and optical filters to capture the detection results of the wearable patch. After processing by algorithms, the user can see the detection result on the smart terminal.

Sweat detection can be used to evaluate training performance and training degree and design appropriate training programs. Cai et al. [112] developed a lactic acid sensor based on electroluminescence using luminol as a signal substance. Hydrogen peroxide produced by lactic acid decomposition under the action of enzymes made a luminous glow, which enabled the sensor to distinguish the intensity of the exercise by detecting lactic acid, and then find the critical point of exercise intensity. The amount of sweating is vital to the body because excessive sweating breaks the electrolyte balance in the body, resulting in dehydration. As shown in Figure 8b, a patch designed to detect sweat rates can collect sweat in chronological order and perform discrete real-time in situ testing [113]. The pigmented tip changed color when saturated with sweat so that the sweat rate and degree of dehydration can be easily determined. At the same time, the patch is also low-cost and can be manufactured on a large scale. Continuous testing of lactic acid in sweat to analyze the lactate threshold, i.e., the transition from aerobic to anaerobic metabolism, could be used to optimize training for athletes, physical exercise for rehabilitation patients and the elderly, and high-intensity individuals such as firefighters [114]. Kim et al. [115] developed a multifunctional sweat platform that measures cortisol associated with stress and glucose, vitamin C, and sweat rates associated with physiology. Sweat rate was measured by the changes of electrical resistivity through electrodes embedded in microchannels in contact with sweat; glucose and vitamin C were monitored by fluorescence; and cortisol concentration was measured by an anti-cortisol antibody (ACA)-AuNPs alternative enzyme-linked immunosorbent assay. In combination with near-field communication, wireless digital tracking monitoring of the above-mentioned markers can be achieved.

Sweat is quickly lost, so it is necessary to store it. The main current solution is to leave sweat storage areas on the sensor patch, which has been confirmed by the study of McCaul et al. [116]. In addition to setting the sweat storage area, Martín et al. [117] used lithography and screen printing to fabricate an epidermal microfluidic electrochemical detection platform for efficient and rapid sweat collection and metabolite detection. As shown in Figure 8c, an electrode system, microfluidic channels, detection reservoirs, and medical tapes formed an efficient natural sweat pump, which allowed the flexible microchip device to be attached to the skin and maintain stable contact with the skin at all times for rapid sweat collection. This design ensured that the detector always had a sufficient volume of sweat for detection while removing the initial contaminating sweat metabolites. The sweat detection mentioned above is based on post-exercise but sweat sensors do not work for people who do not sweat or have low sweat volume, especially patients. The current means used is the artificial stimulation of sweating, such as delivering the introduction of pilocarpine ions into the skin via electric current. What most sweat sensors can achieve is sweat detection, few sensors can actively intervene, and we hope to develop more sensors with closed-loop control functions in the future.

### 4.2. Blood

Blood contains a large number of substances related to human health. In medicine, blood tests are used as one of the gold standards for health diagnosis. Traditionally, blood tests are performed by invasive means, i.e., by drawing a certain amount of blood. Newly developed devices allow non-invasive testing. These devices are mainly used to test blood oxygen, blood glucose, and blood pressure.

**Figure 9:**
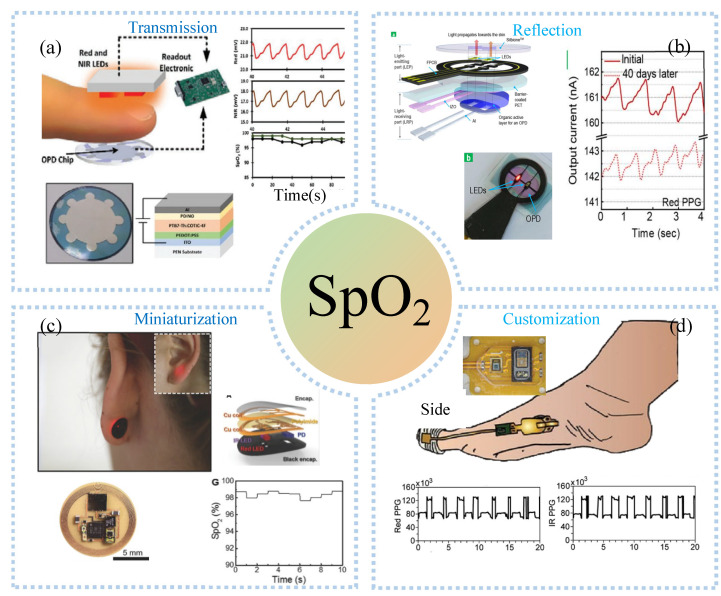
Blood detection: (**a**) transmissive PPG sensor at the fingertip and provides reliable blood oxygen monitoring. Reprinted with permission from Refs. [99]. Copyright 2022 John Wiley and Sons, (**b**) reflective PPG sensor at the fingertip and provides reliable blood oxygen monitoring. Reprinted with permission from Refs. [119]. Copyright 2021 American Chemical Society, (**c**) miniaturized PPG sensor monitoring blood oxygen in the earlobe. Reprinted with permission from Refs. [120]. Copyright 2016 John Wiley and Sons, (**d**) customized PPG sensors at the toes and provide reliable blood oxygen monitoring while walking. Reprinted with permission from Refs. [121]. Copyright 2020 John Wiley and Sons.

Pulse oximetry is the most commonly used commercial blood oxygen testing device, which requires accurate mounting and wearing of the device during use. Meanwhile, large sizes and high-power consumptions make it impossible to use pulse oximetry for real-time, remote blood oxygen testing. The PPG-based sensor consists of a light-emitting diode and a photodetector (PD) that allows the detection of the blood oxygen process in opaque tissue sites. PD and light-emitting diodes were placed in opposition or side-by-side for detecting transmitted (Figure 9a) or reflected (Figure 9b) light from biological tissues, which are converted into an electrical signal output. Compared to rigid PPG sensors, flexible organic photodetector (OPD) is skin-adapted and comfortable. To this end, Bae et al. [118] assembled μLEDs, organic PDs, and heaters embedded in PDMS to make a composite device with 50% strain and strong emissions, which was used to monitor vital signs such as heart rate, deep breathing, cough, and blood oxygen saturation. Organic OPD was made by spin coating PEDOT: PSS on ITO for receiving the light of μLED. The heaters made of spin coated AgNWs on PDMS substrates can widen blood vessels. Compared with the transmissive type, the reflective PPG sensor can be used for blood oxygen detection in all parts of the body. Lee et al. [119] demonstrated hybrid reflection-type (R-type) PO, which contained inorganic LEDs and a wrap-around OPD (Figure 9b). OPD monitors blood oxygen levels by receiving reflected light from blood vessels. In addition, the hybrid POs can reduce the power by by alternately turning on red and near-infrared leds. Combining near-field communication and flexible electronics, Kim et al. [120] developed a small flexible wearable pulse oximeter for monitoring blood oxygen at the earlobe, as shown in Figure 9c. The sensor was only the size of a fingernail and can run continuously for up to 3 months. The motion artifacts of the sensor come mainly from poor contact during motion. Another way to miniaturize is to remove additional components. With the advantages of 3D printing customization and low-cost manufacturing, Abdollahi et al. [121] used free-form reversible embedding (FRE) 3D printing to customize a dedicated pulse oximeter for patients, as shown in Figure 9d, including red and infrared LEDs, PD, pressure sensors, and flexible circuit boards. Blood oxygen monitoring at the toes at rest and sitting was similar to that obtained with commercial products. Nonetheless, they also found that the blood volume and contact between skin and sensor were constantly changing during walking due to the constant change of pressure, making it difficult for the sensor to obtain an accurate blood oxygen value. The motion artifacts of the sensor come mainly from poor contact during motion. Another way to miniaturize is to remove additional components. For example, Han et al. [122] proposed an ambient light oximeter, which used various kinds of ambient light, getting rid of the demand for LEDs. They combined spectral filters and OPDs to detect SPO_2_ on the index finger in different lighting conditions, and its performances were consistent with commercial oximeters.

In addition to blood oxygen, blood sugar is also an important health marker in the blood. Invasive methods are the current gold standard for blood glucose testing. Even blood glucose meters detect blood glucose electrochemically, which is an invasive or minimally invasive method. Non-invasive optical sensing provides a better choice for blood glucose detection. The current PPG sensors that can be used for blood glucose detection are predominantly based on near-infrared and Raman spectroscopy. Joshi et al. [123] proposed a NIR serum glucose level monitoring line using absorption- and reflection-based dual NIR spectroscopy, and a calibrated machine learning model. The first channel utilized a transmission configuration to measure the absorption of the LEDs at 1300 nm, and the second and third channels involved the absorption and reflection spectra of the LEDs at 940 nm. Serum glucose prediction was performed using deep neural networks and polynomial regression models. Compared with other NIR measurement techniques, the device showed a highly accurate prediction of serum glucose. In addition to the above blood health indicators, testing equipment for other substances such as blood lactate has also appeared. Yang et al. [124] developed a microwave-range electromagnetic-based blood lactate sensor, which was placed on the arms and legs to detect blood lactate, using invasive blood lactate testing as a standard. They found that the detection results of the developed sensor had a good correlation (R^2^ = 0.78), and the error was 13.4% in the range of 0~12 mmol/L. NIR-based blood lactate has also been developed, such as the BSX Insight lactate prediction system. The system can detect the concentration of lactate in the blood during exercise to predict the lactate threshold.

### 4.3. Interstitial Fluid

As one of the components of body fluids, interstitial fluid occupies about 60–70% of body fluids, and its composition is mostly determined by the types of cells around it. The detection results of its composition and biophysical properties can be used to judge the health status of surrounding cells, thereby diagnosing cytopathology. The fluid has many chemicals similar to blood, such as glucose, lactate, cortisol, and urea. Therefore, the content of the same markers in the blood can be judged by monitoring the health markers in the interstitial fluid.

**Figure 10:**
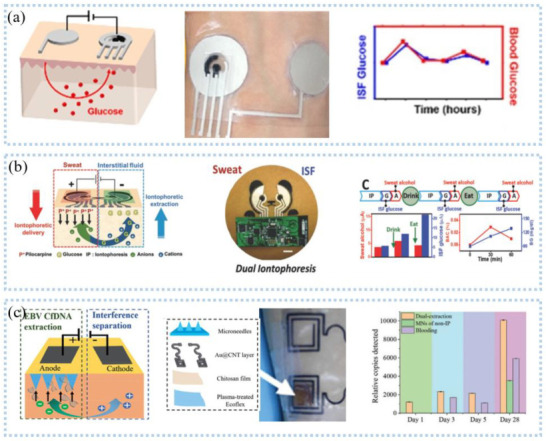
Interstit7401 fluid detection−reverse ion osmosis: (**a**) schematic diagram of reverse parti2552cle osmosis (left), patch (middle) and glucose monitoring (right). Reprinted with permission from Ref. [125]. Copyright 2021 American Chemical Society, (**b**) Schematic diagram of sweat and glucose extraction (left), panda head sensor system (middle), and sweat and glucose monitoring (right). Reprinted with permission from Ref. [128]. Copyright 2018 John Wiley and Sons, (**c**) Schematic diagram of microneedle and reverse ion osmosis principle (left), physical object (middle), and glucose monitoring (right). Reprinted with permission from Ref. [129]. Copyright 2019 John Wiley and Sons.

Reverse ion-osmosis (RI) extracts substances through the potential difference between two electrodes on the skin (Figure 10a), and charged ions migrate directionally under the action of an electric field, such as sodium ions that accumulate toward the cathode [125]. The cathode with a large number of sodium ions promotes the gradient permeation of water, and the neutral species in the interstitial fluid also permeate with it. Bandodkar et al. [126] demonstrated a fully printed tattoo-based temporary glucose sensor. A pair of reverse iontophoresis electrodes, a reference electrode, and a working electrode were printed on the substrate by a screen-printing process, and the working electrode was modified with lactate oxidase. Blood glucose testing of the test subjects one hour after eating revealed elevated postprandial glucose levels. The two-dimensional array sensor design can be used for calibration-free and accurate glucose detection. Lipani et al. [127] developed a graphene-based array glucose sensor utilizing interstitial fluid transdermal RI for glucose extraction. The device consisted of a 4 × 4 array of sensors that allowed for non-invasive and path-selective glucose detection. Monitoring glucose before and after lunch and snacks revealed that the arrayed sensors produced well-matched readings, which confirmed their ability to accurately monitor blood glucose. Kim et al. [128] fabricated a panda electronic tattoo patch sensor by screen printing, which could simultaneously detect the sweat extract of the anode and the ISF extract of the cathode, as shown in Figure 10b. The detection of glucose and alcohol after food consumption and alcohol consumption correlated well with commercial blood glucose meters and breathalyzer devices.

The extraction of interstitial fluid by the RI technique generally takes 5–10 min and the extraction rate is slow, which makes the technique incapable of real-time detection of interstitial fluid. The development of microneedle manufacturing technology has broken the defect that reverse iontophoresis technology is difficult to detect interstitial fluid in real-time. Teymourian et al. [129] developed a microneedles device for continuous real-time detection of ketone bodies, which can be detected within the lower limit (50 μm). Experiments confirmed the device’s ability to detect ketone bodies, suggesting that it can be used for real-time continuous interstitial fluid monitoring of diabetic ketosis and ketoacidosis. To rapidly capture important biomarkers, a wearable epidermal system (Figure 10c) combining reverse ion introduction and MNs was developed [130]. This system can isolate cell-free DNA targets from ISF in less than 10 min while having a maximum capture efficiency of 95.4%. Ciui et al. [131] integrated the bandage sensor and the microneedles sensor on the flexible PCB board and realized the wearable skin melanoma detection through wireless human-computer interaction. Integrating unmodified and tyrosinase-modified carbon paste microneedles electrodes onto the same sensor array patch enables simultaneous independent enzymatic-amperometric and non-enzymatic volumetric dual mode sensing of L-Dopa. The device can be used to continuously monitor the anti-Parkinson’s drug L-Dopa, which facilitates the movement towards Parkinson’s disease management.

Pu et al. [132] utilized ultrasound to improve skin permeability and extracted interstitial fluid under vacuum. They connected three electrodes to a microfluidic chip in which the working electrode was made of graphite and AuNPs to detect glucose. This method, also known as sonography, works on the principle that ultrasound induces cavities, increasing the porosity of the skin, and skin permeability increases when vacuum pressure is combined with ultrasound. Although a certain number of micropores are generated in the skin during the detection process, it does not cause damage to the skin. Soto et al. [133] designed a flexible transdermal tattoo patch with microspheres on the patch enhancing the ability of drug delivery, which can be used for various treatments and detoxification in the future in combination with the penetrating needle-free ultrasonic microsphere process. The detection of interstitial fluid has developed rapidly, and it is also possible to search for the use of interstitial fluid for drug delivery to treat certain diseases. Yet, few wearable sensors that can be retrieved for detection and treatment are capable of active closed-loop intervention.

## 5. Multi-Signals

### 5.1. Multidimensional Signal

**Figure 11:**
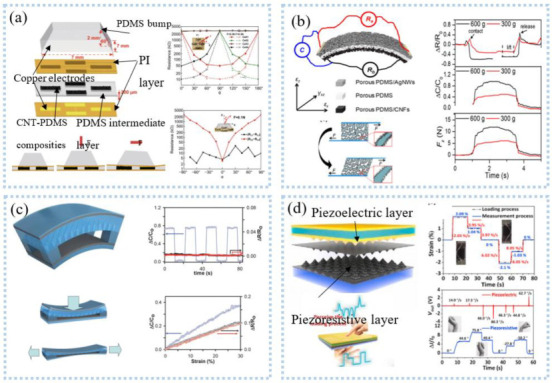
Multi−dimensional signal detection: (**a**) the conductive porous nanocomposites under shear deformation structure schematic diagram of multi−axial force sensor based on four−unit combination (upper left), and schematic diagram of triaxial force monitoring (lower left), resistance change of each cell to angles (right). Reprinted with permission from Ref. [137]. Copyright 2019 Springer Nature, (**b**) conceptual design of a unitized sensor for simultaneous measurement of multiple forces (upper left), deformation mode (lower left), corresponding relative resistance (right). Reprinted with permission from Ref. [139]. Copyright 2020 American Chemical Society, (**c**) multiaxial force detection based on capacitance and friction. Reprinted with permission from Ref. [143]. Copyright 2014 John Wiley and Sons, (**d**) schematic illustrations of the multifunctional dual−mode pressure sensor (upper left), capture of dynamic and static forces (lower left), comparison of the complex bending process between the actual and measured processes (upper right), human motion decoder (lower right). Reprinted with permission from Ref. [144]. Copyright 2020 Elsevier.

Compared with uniaxial signals, multi-axial signals are more common, such as touch, multi-axial force, and position information. Vertically staggered CNT fibers on Ecoflex substrates enabled the detection of multi-axis human motion [134]. Zhang et al. [135] placed graphene fabrics in the shape of roses with 120° between them, creating sensors for multidirectional force detection, which can detect strain and force in different directions. The detection of three-dimensional information also adopted a four-unit combination [136], as shown in Figure 11a. The protruding structure at the top of the sensor can convert the mechanical force into stress, so that the resistance of the four units changes, to calculate the normal force and shear force [137]. Another similar one is the 3D flexible tactile sensor developed by Wattanasarn et al. [138], which consisted of four identical copper coils encapsulated in PDMS. When a force was applied to the protruding structure on the upper end of the sensor, the deformation of the coil made the induced voltage change, realizing the detection of 3D tactile sensation.

Unlike the single detection mode above, Peng et al. [139] developed multimodal sensors for measuring multidimensional forces. Porous PDMS was used as the intermediate medium, and the upper and lower electrodes were respectively made of porous PDMS/AgNWs and porous PDMS/CNFs to form an asymmetric sandwich structure, as shown in Figure 11b. The sensor can detect normal pressure, transverse tension, and transverse shear force at the same time. This structure was exquisite, but the detection of force in one direction was easily disturbed by other directions. Inspired by human skin, sensors with highly anisotropic structures and different responses to stimuli in different directions can be used to discriminate 3D stimuli. Based on this, Chen et al. [140] fabricated stretchable 3D anisotropic piezoresistive sensors. The Au/PDMS micro-dome on the upper layer was used to detect the Z-axis force. The interlocked PUGA/PDMS hard micro-dome structures responded to the X and Y axes, respectively, without interfering with each other. The sensor can achieve direction discrimination in plane strain with a selectivity of 3.68 and a sensitivity of 0.62 kPa^−1^. Integrating strain sensors and clothing created a smart sleeveless shirt that measures the kinematic angle of the torso relative to the pelvis [141]. Tavassolian et al. [142] developed a modular and size-adjustable inductive sensor to measure the angular change of multiaxial hips during running. Using an optical motion capture system as the gold standard, the accuracy is as high as R^2^ = 0.98. In the process of human monitoring, simultaneous dynamic and static monitoring can provide more information to accurately decode human movements. As shown in Figure 11c, a sensor combing capacitance and friction can differentiate mechanical stimuli, such as normal pressure, lateral strain, bending, and vibration [143]. The amplitude of the stimulus can be obtained by detecting the change in capacitance, while the frequency and direction can be obtained from the friction signal. The result was that the stimulus can be identified from both the capacitance signal and the friction signal. In another similar example, Qiu et al. [144] proposed a dual-mode pressure sensor with interlocking piezoelectric and piezoresistive films, as shown in Figure 11d. Piezoresistive films can monitor the duration of force, and piezoelectric films can monitor the rate of change and direction of force, which provided rich information for analyzing human actions, such as applying the direction, frequency, and amplitude of the stimulus. The researchers successfully used the dual-mode pressure sensor to decode complex human movements.

Multidimensional information detection has been applied to health assessments. For example, four capacitive sensors were attached to the back L5 of the human body to detect the bending and twisting of the torso [145]. The bending and twisting of the trunk are the main factors of occupational low back pain, and the angle analysis of the low back movement can well monitor the back movement to prevent low back pain. Later sensors for monitoring surface roughness, surface texture, and slippage were also developed [146]. Braille has an uneven surface with convex and concave, which can be well recognized by identifying the surface texture. Permanent magnet nanocomposites made of PDMS and iron nanowires are used to read Braille [147]. The external force changed the magnetic field in the artificial fiber hair made of PDMS and iron nanowires, which changed the resistance and improved the sensitivity. Experiments have shown that Braille can be recognized well, which could help blind people achieve better reading and pronunciation in the future.

### 5.2. Multimode Signal

As mentioned above, various sensors for health information detection have been developed. There are various kinds of information on the human body. It is ideal to be able to detect multiple information simultaneously with one sensor. Several studies have reported sensors with multiple stimulus responses for the detection of multiple signals. Research shows that piezoresistive-based strain sensors can also be used for the detection of bioelectrical signals. For example, highly conductive multilayer graphene nanosheet film can be made into a tattoo dry electrode to accurately detect bioelectrical signals, such as ECG and EMG [148]. Meanwhile, the serpentine structure can accurately detect wrist pulse and breathing and can be reused more than 2000 times. The work done by Kim et al. [22] demonstrated that multiple signals could be simultaneously detected by a single sensor, and they developed a wireless, soft, and comfortable ECG detection system by integrating cardiac electrodes and wireless detection circuits on a flexible substrate. The system can realize real-time and remote wireless monitoring of ECG, heart rate, and respiratory rate at the same time by processing the detected ECG signal. Selecting Au thin films as electrodes, an electronic tattoo (1.5 μm) with a serpentine structure was fabricated on PET by cutting and pasting, which could respond to temperature and bioelectrical signals [149]. It was attached to the human skin and allowed accurate measurements of ECG, temperature, and skin hydration. Some studies have shown that heart rate and breathing rate can also be derived from ECG signals. The same device responds to multiple stimuli simultaneously; Fu et al. [150] proposed a new wearable pulse monitoring system based on thermal sensing, which had been applied to detect pressure and temperature stimuli. They placed two sensors on the wrist that detected pulse waves, PWV, and skin temperature.

**Figure 12:**
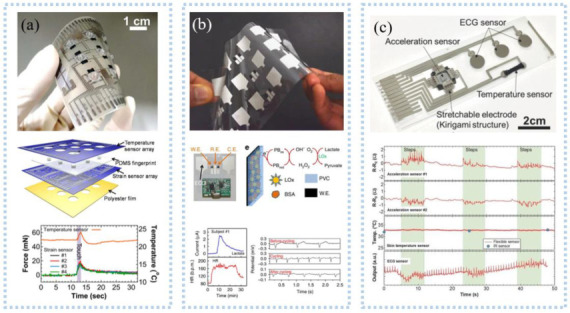
Multimode signal detection: (**a**) Picture of a 3 × 3 array e−skin (upper), schematic for each layer of the e−skin device (middle), results of real−time measurements of the temperature and strain sensors (lower). Reprinted with permission from Ref. [152]. Copyright 2014 John Wiley and Sons, (**b**) an array of printed Chem−Phys flexible patches (upper), image of a Chem–Phys patch along with the wireless electronics (middle), results of the real-time lactate concentration and the heart rate (lower). Reprinted with permission from Ref. [153]. Copyright 2016 Springer Nature, (**c**) a fabricated sensor sheet, showing the positions of the integrated sensors (upper) and real−time results for simultaneous measurement of human motion, skin temperature, and heart rate. Reprinted with permission from Ref. [155]. Copyright 2017 John Wiley and Sons.

Various sensors are integrated to detect multiple signals. For example, a novel layered sensor consisting of stainless-steel film, PVDF film, and the photoelectric sensor was fabricated to simultaneously detect EMG, MMG, and oxygen consumption at the same location [151]. The superimposed sensors remove the mutual interference. Such sensors are also used for 3D tactile and temperature measurements [152]. The strain sensor and temperature sensor were layered on a PET flexible substrate by screen printing and then laminated into one, as shown in Figure 12a. Triaxial force and temperature could be detected simultaneously. Triaxial forces such as tactile force and slip force can be measured by a PDMS strain sensor with a similar fingerprint structure. The temperature sensor consists of CNT-PEDOT: PSS and Ag electrodes and can measure temperature at 0.25%/°C sensitivity. Imani et al. [153] prepared a wearable hybrid sensing system by screen printing, which could realize the simultaneous detection of physical and chemical parameters, as shown in Figure 12b. The three-electrode lactate electrochemical sensor and the heart electrode were printed on the same flexible substrate to make a 4 × 3 array, which could realize the simultaneous detection of lactate and ECG and exhibited a lactate sensitivity of 96 nA/mM. A wearable device for simultaneous detection of sweat pH and the temperature has also been developed [154]. Compared with the above two sensors, Yamamoto et al. [155] fabricated a planar multifunctional health sensor that can detect three signals simultaneously, as shown in Figure 12c. The acceleration sensor, temperature sensor, and ECG sensor of the kirigami structure were integrated into PET by screen printing, realizing the simultaneous detection of human motion, skin temperature, and ECG signal. The fabric is comfortable to wear, and the integration of conductive fabric and motion sensor into the textile electrode enabled simultaneous detection of ECG and motion. A tailored e-textile conformal suit for large-scale and multimodal physiological detection [156], which integrated acceleration sensors, temperature sensors, and wireless transmission modules to detect temperature, respiration, and heart rate during exercise detection. The laundry performed as before after washing and had good robustness.

Multidimensional signals are more advantageous than single signals for assessing exercise and health. Kwon et al. [157] integrated PPG sensors and acceleration sensors on a flexible circuit to make a wearable hybrid electronic device. The elastomeric Ecoflex gel was used as the encapsulating material to allow the device to adhere stably to the skin and to more accurately detect heart rate and pulse during exercise than a rigid wristband system. The acceleration sensor can be used by boxers in a variety of fights to assess boxing speed and intensity. A multi-node highly stretchable and conformable matrix network (SCMN) consisting of six different types of sensors was used for the detection of temperature, in-plane strain, relative humidity, UV light, magnetic fields, pressure, and proximity [158]. An intelligent prosthesis for detecting temperature and pressure was designed through a personalized combination that helps disabled patients regain force-sensing functions for grasping control, object manipulation, and temperature sensing. The detection of health indicators has been widely used in health assessments. Wearable devices with health detection, diagnosis, and treatment functions can achieve closed-loop monitoring and adjust health. Son et al. [159] designed a multifunctional wearable device for the diagnosis and treatment of movement disorders. Strain sensors, temperature sensors, TiO2NMRRAM arrays, resistive heaters, and data memory were packaged on an elastic colloidal patch. Strain sensors can detect different tremor frequencies, a powerful method for monitoring and diagnosing movement disorders. The detected signals were processed through data to diagnose and identify movement disorders and trigger therapy. At this time, the drug was delivered through the transdermal layer, and the resistive heater reduces the physical bond between the nanoparticles and the drug, enlarges the pores, and increases the drug delivery rate. The device realized the function of integration of diagnosis and treatment and overcame the limitation that most current devices can only perform detection without treatment.

## 6. Summary and Outlook

Wearable sensors have been rapidly developed and play an important role in health and sports. This paper presents a review of wearable sensors for continuous monitoring of vital signals in health and exercise. These include bioelectrical signals (ECG, EEG, and EMG), biophysical signals (inertia, body motion, heart rate, and pulse), biochemical signals (sweat, blood, and interstitial fluid), and multi-signals (multidimensional and multimodal signals). As the research progresses, these wearable devices are small in size, high in accuracy, flexible, stretchable, biocompatible, low in production cost, real-time continuous monitoring, etc., and have wide application prospects in various medical, rehabilitation, and motion analysis fields. Wearable devices containing flexible sensors, wireless communication, and power supply are more practical in the field of health and sports monitoring. These devices can achieve wireless and remote real-time monitoring of vital signals and play an important role in disease diagnosis and prediction, sports assessment, and injury warning. In addition, combined with artificial intelligence algorithms and IoT technology, these real-time monitoring data can provide real-time alerts to users and help healthcare professionals make treatment plans. With visualization software and displays, users can get a real-time view of their personal health.

Thanks to advanced manufacturing, flexible electronics, the internet of things, and artificial intelligence algorithms, there has been significant development in sensing devices with flexible, wearable, portable, and real-time monitoring capabilities. However, flexible wearable sensing devices still face challenges and limitations in terms of signal monitoring accuracy, continuous monitoring, multi-signal monitoring, IoT and cloud, and prediction and diagnosis, especially in commercialization. To this end, the above issues must be addressed to realize the full potential of flexible wearable sensing devices, as follows.

Accuracy: The accuracy and reliability of signals are mainly affected by power lines [44] and motion artifacts [157]. For power lines, the distance between the sensor and the data processing module is as short as possible so that electromagnetic interference caused by too-long power lines can be avoided. For motion artifacts, ultra-thin self-adhesive sensors are the future choice, especially relying on van der Waals forces to form a strong fit with the skin [22,23,24], while also having the advantages of lightweight and non-sensitization. Motion artifacts mainly originate from the gap between the skin and the sensor due to motion. Choosing a base with the same modulus as the skin can enhance the compliance between the sensor and the skin [24,60]. Combining the above, the development of a signal that can be used to monitor accurately during motion and long-term use is highly anticipated.

Multimodality: Health often corresponds to multiple factors, and it is difficult to accurately diagnose and predict health by relying on a single signal [149,151,154]. Therefore, new sensors capable of monitoring multiple signals are needed to find the root cause, which is especially important in the diagnosis and prediction of diseases, such as heart disease. Integrating different sensor components into a single sensor is a good option [66], and of course, split manufacturing is also available. However, a challenge will be the fabrication. In addition, one sensor is capable of responding to multiple stimuli, which is particularly common in strain sensors. This problem may be solved by the design of the structure and sensing mechanism.

Continuity: One issue that must be addressed for long-term, continuous monitoring is energy [160]. In particular, the increase in monitoring targets and processing requirements further leads to increased power consumption and expanded energy requirements. This problem can be effectively addressed by optimizing power management, minimizing power consumption processing, or increasing the power supply. Self-powered sensors can reduce power consumption very well. In addition, energy is harvested from the body itself for energy supply, such as piezoelectric devices, thermoelectric devices, solar devices, and electrochemical devices [17,78,79]; however, this energy is often relatively small. Therefore, it is highly desirable to develop more advanced energy supply strategies in future wearable devices for sports and health detection.

Cloud: Cloud-based vital signal detection systems can be used for health diagnosis and prevention, which is important for the elderly. However, current cloud-based monitoring systems still have the following problems: real-time interaction between professionals and users, real integration of physical and information systems of physical and information systems, poor warning accuracy, and full life-cycle monitoring [161]. The current problem of early warning accuracy can be effectively solved by improving algorithms and adding expansion ports; however, this still cannot solve the fundamental problem. Data fusion may be effectively solved by the digital twin [161]. In addition, the communication transmission distance is short, and data loss, low sampling frequency, and power consumption are too high, especially when in motion [1]. Data must be processed after transmission before it can be used [18], and it is difficult for general smart devices to support artificial intelligence algorithms. Uploading data to the cloud, with the help of offsite servers for intelligent data processing, will facilitate real-time monitoring, diagnosis, and early warning of health and exercise [36,47,51,64]. Meanwhile, the data in the cloud can be connected to hospitals, community health centers, and smart terminals of family members.

Prediction and diagnosis: Although relying on AI algorithms and multimodal sensors can diagnose and predict diseases, however, the diagnostic and predictive capabilities based on big data and AI algorithms are still underdeveloped. The key to this lies in the analysis of the data, which often still relies on medical professionals and is not useful for users [1,2,15]. In addition, there are still very few diseases for which diagnosis and prediction are currently possible, which is also true for sports. Collaborating with healthcare professionals to build models for disease prediction and diagnosis [2], while making wearable devices with more signal monitoring capabilities, is the way forward. Thus, wearable devices with multiple signal monitoring capabilities and new diagnostic and predictive AI algorithms are expected to enable more accurate diagnosis and prediction.

## Data Availability

The study did not report any data.
